# Gut dysbiosis patterns in CVID patients with noninfectious complications observed in a germ-free mouse model through fecal microbiota transplantation

**DOI:** 10.70962/jhi.20250040

**Published:** 2025-04-23

**Authors:** Joud Hajjar, Anita Y. Voigt, Margaret E. Conner, Alton G. Swennes, Stephanie Fowler, Chadi Calarge, Danielle D. Mendonca, Dominique Armstrong, Chen-Yen Chang, Jolan E. Walter, Manish J. Butte, Tor Savidge, Julia Oh, Farrah Kheradmand, Joseph F. Petrosino

**Affiliations:** 1 The William T. Shearer Center for Human Immunobiology at Texas Children’s Hospital, Houston, TX, USA; 2 Department of Pediatrics, Section of Immunology, Allergy and Retrovirology, Baylor College of Medicine, Houston, TX, USA; 3 The Jackson Laboratory for Genomic Medicine, Farmington, CT, USA; 4 Department of Dermatology, Duke University School of Medicine, Durham, NC, USA; 5 Departments of Molecular Virology and Microbiology & Education, Innovation and Technology, Baylor College of Medicine, Houston, TX, USA; 6 Office of Comparative Medicine and Department of Pathology, School of Medicine, University of Utah, Salt Lake City, UT, USA; 7 Center for Comparative Medicine, Department of Molecular Virology and Microbiology, Baylor College of Medicine, Houston, TX, USA; 8 Menninger Department of Psychiatry and Behavioral Sciences and Department of Pediatrics, Baylor College of Medicine, Houston, TX, USA; 9 Department of Medicine, Baylor College of Medicine, Houston, TX, USA; 10 Interdepartmental Program in Translational Biology and Molecular Medicine, University of South Florida, Tampa, FL, USA; 11 Division of Allergy/Immunology in Department of Pediatrics, University of South Florida, Tampa, FL, USA; 12 Division of Allergy/Immunology in Department of Pediatrics, Johns Hopkins All Children’s Hospital (JHACH), St. Petersburg, FL, USA; 13 Departments of Pediatrics and Microbiology, Immunology, and Molecular Genetics, University of California, Los Angeles, Los Angeles, CA, USA; 14 Department of Pathology & Immunology, Baylor College of Medicine, Houston, TX, USA; 15 Texas Children’s Microbiome Center, Department of Pathology, Texas Children’s Hospital, Houston, TX, USA; 16 Biology of Inflammation Center, Baylor College of Medicine, Houston, TX, USA; 17 Center for Translational Research On Inflammatory Diseases (CTRID), Michael E. DeBakey Department of Veterans Affairs, Houston, TX, USA; 18 Alkek Center for Metagenomics and Microbiome Research, Department of Molecular Virology and Microbiology, Baylor College of Medicine, Houston, TX, USA

## Abstract

Patients with common variable immunodeficiency (CVID) who develop noninfectious complications (NIC) have worse clinical outcomes than those with infections only (INF). While gut microbiome aberrations have been linked to NIC, reductionist animal models that accurately recapitulate CVID are lacking. Our aim in this study was to uncover potential microbiome roles in the development of NIC in CVID. We performed whole-genome shotgun sequencing on fecal samples from CVID patients with NIC, INF, and their household controls. We also performed fecal microbiota transplants from CVID patients to germ-free mice. We found potentially pathogenic microbes *Streptococcus parasanguinis* and *Erysipelatoclostridium ramosum* were enriched in gut microbiomes of CVID patients with NIC. In contrast, *Fusicatenibacter saccharivorans* and *Anaerostipes hadrus*, known to suppress inflammation and promote healthy metabolism, were enriched in gut microbiomes of INF CVID patients. Fecal microbiota transplant from NIC, INF, and their household controls into germ-free mice revealed gut dysbiosis patterns only in recipients from CVID patients with NIC, but not in those from INF CVID or household controls recipients. Our findings provide a proof of concept that fecal microbiota transplant from CVID patients with NIC to germ-free mice recapitulates microbiome alterations observed in the donors.

## Teaser

Hajjar et al. identify distinct gut microbiome profiles in CVID patients with noninfectious complications and establish a novel germ-free mouse model using fecal microbiota transplant. This model recapitulates human dysbiosis and provides a platform to explore microbiome-driven immune dysregulation in CVID.

## Graphical Abstract

**Figure:**
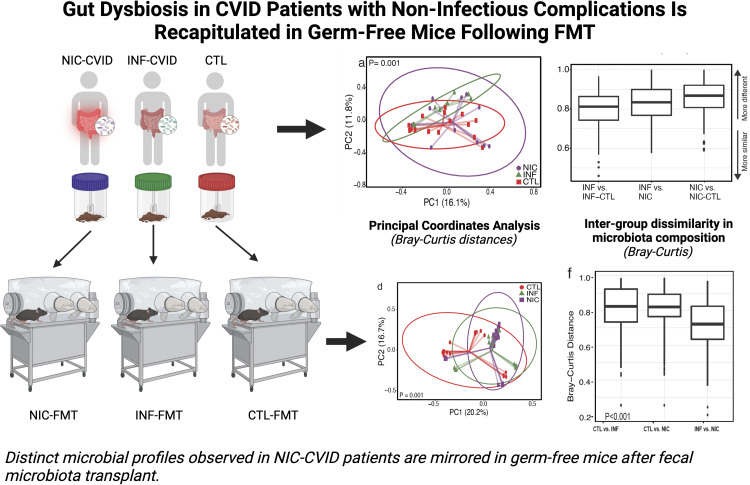

## Introduction

Common variable immunodeficiency (CVID) is the most prevalent treatable inborn error of immunity in adults (1, 2). It is characterized by low immunoglobulin (Ig) levels (IgG, IgA, and/or IgM) and recurrent infections due to B cell defects (3). Clinically, CVID manifests in two broad phenotypes: infections only (INF) and noninfectious complications (NIC), which include autoimmune and autoinflammatory disorders (1, 4, 5). Nearly 60% of CVID patients develop NIC, which manifests as cytopenia, inflammatory bowel disease-like disease, chronic lung disease, and lymphoproliferation (1, 6, 7, 8, 9). In addition, NIC-CVID patients have a significant increase in morbidity and mortality compared with INF-CVID patients (10, 11, 12). Thus, there is a pressing need to improve our understanding of NIC-CVID.

Several recent studies have suggested involvement of the gut microbiome in CVID-associated immune dysregulation. Specifically, bacteria and their associated products translocate across “leaky” gut epithelium into systemic circulation, as evidenced by the detection of circulating lipopolysaccharide (LPS) or bacterial DNA (13, 14, 15). Furthermore, LPS activates an immune response through the recognition of microbe-associated molecular patterns (16, 17), releasing pro-inflammatory cytokines (18), and those mechanisms may have implications in CVID. Other clinical studies have also shown that the gut microbial composition is altered in CVID patients (i.e., dysbiosis), particularly in NIC-CVID (14, 19, 20). Furthermore, the 16S ribosomal RNA (16S rRNA) gene sequencing data from CVID patient stool samples showed lower within-sample taxonomic diversity (i.e., alpha diversity) compared with controls (CTLs) (19, 21). Reduced alpha diversity and increased circulating LPS concentration are also more common in NIC-CVID compared with INF-CVID patients, suggesting that the translocation of certain bacteria may be implicated in immune dysregulation observed in NIC-CVID (14). In fact, IgG replacement reduces circulating LPS concentrations, suggesting it may reduce gut bacterial translocation (13) or that polyclonal IgG blocks LPS activity in other ways. Additionally, when mucosal integrity is disrupted, some pathobionts, such as *Acinetobacter baumannii*, induce inflammation by triggering mucosal intestinal macrophages to produce inflammatory cytokines (22). However, it remains unclear if the gut microbiome in NIC-CVID patients is distinct from that in INF-CVID patients and whether NIC-CVID gut dysbiosis can be recapitulated in animal models. Additionally, microbiome diversity, enrichment, and the specific taxa linked to CVID phenotypes remain unclear.

To address these questions, we examined the gut microbiome composition in NIC-CVID and INF-CVID patients, as well as their household CTLs. First, we established the baseline composition of the INF-CVID and NIC-CVID microbiomes at species-level resolution. Then, we comprehensively assessed the gut microbiome using metagenomic whole-genome shotgun sequencing (mWGS) from 11 CVID patients (6 NIC-CVID and 5 INF-CVID) and their available household CTLs (*n* = 8, with one healthy CTL serving as a CTL for 2 CVID patients, who were a mother and a son). Finally, because CVID is a rare disease with no widely accepted animal models, we performed fecal microbiota transplant (FMT) from these CVID patients and household CTLs into germ-free (GF) C57BL/6J mice to assess the feasibility of modeling CVID gut dysbiosis in mice.

## Results

### Gut microbiome alpha diversity is comparable between NIC-CVID and INF-CVID patients, as well as their household CTLs

**Figure 1.**
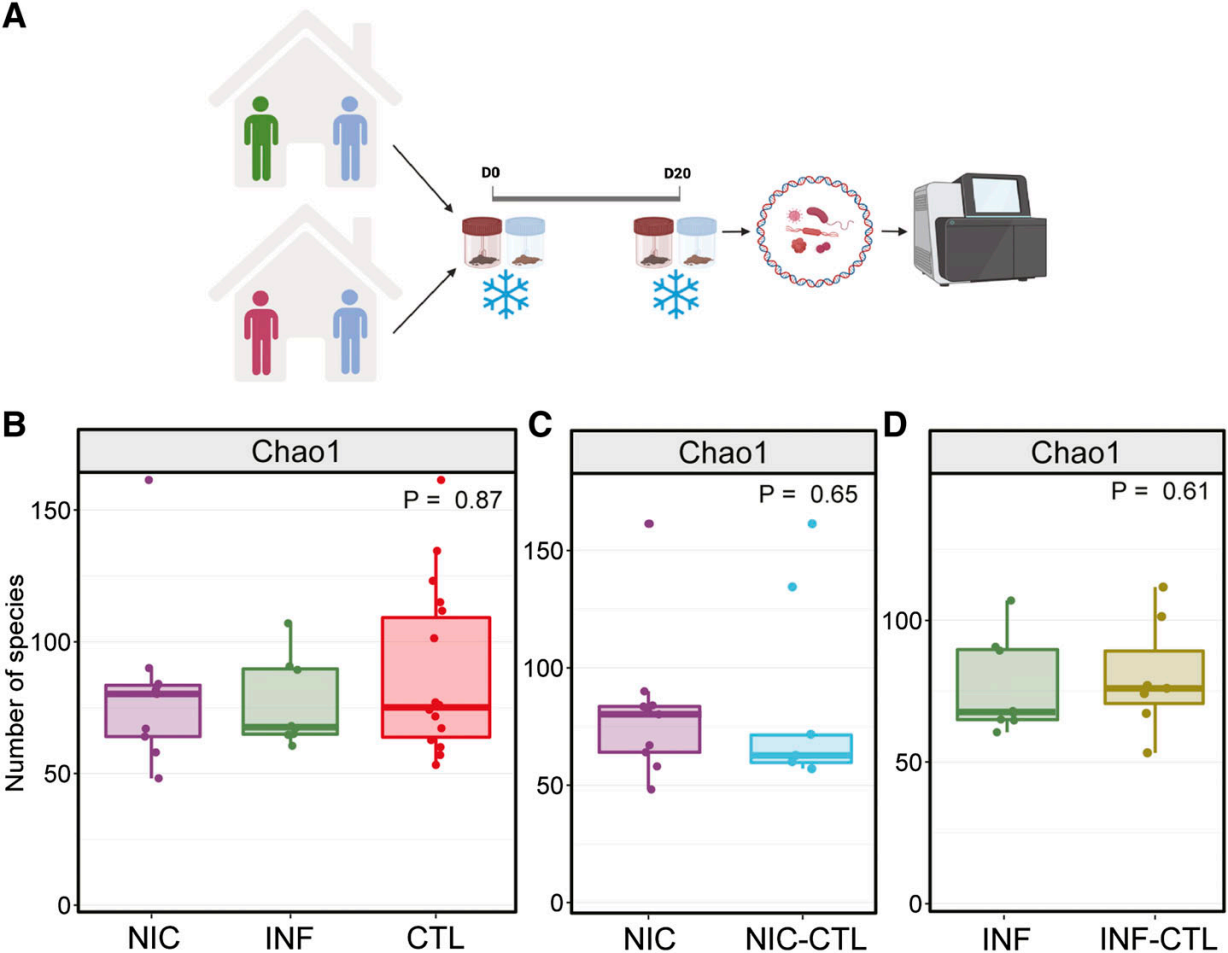
**Richness of the gut microbiome did not significantly differ between CVID patients and their household CTL. (A)** Schematic of the study design for sample collections from CVID patients and household CTL. A total of 11 patients with CVID (6 NIC-CVID and 5 INF-CVID) along with their household CTLs were included. Each patient and CTL provided 2 samples, 20 days apart. Stool samples were collected at home, freshly frozen, and then shipped on ice. **(B)** Microbial alpha diversity is not statistically significantly different between NIC-CVID patients, INF-CVID patients, and household CTLs. **(C and D)** No significant differences between NIC-CVID and their matched household (C) CTL (NIC-CTL) or (D) INF-CVID with their household CTLs (INF-CTL). The analysis was performed by Kruskal–Wallis H test in B and by the Mann–Whitney *U* test in C and D. Panel A was created with https://BioRender.com. Total of 6 NIC, 4 NIC-CTL, 5 INF, and 4 INF-CTL.

Greater diversity within each sample, known as alpha diversity, is often associated with a stable microbiome and healthy metabolism (23, 24). To determine the effect of CVID on gut microbiome richness and diversity, we performed mWGS on the gut microbiome of NIC-CVID and INF-CVID patients, as well as their household CTLs (Fig. 1 A). Microbial alpha diversity did not differ significantly between NIC-CVID, INF-CVID, and their household CTLs (Fig. 1 B). Additionally, no significant differences in alpha diversity were observed between NIC-CVID patients and their matched household CTLs or between INF-CVID patients and their household CTLs (Fig. 1, C and D).

### NIC-CVID and INF-CVID patients exhibit dissimilar gut microbiome composition

**Figure 2.**
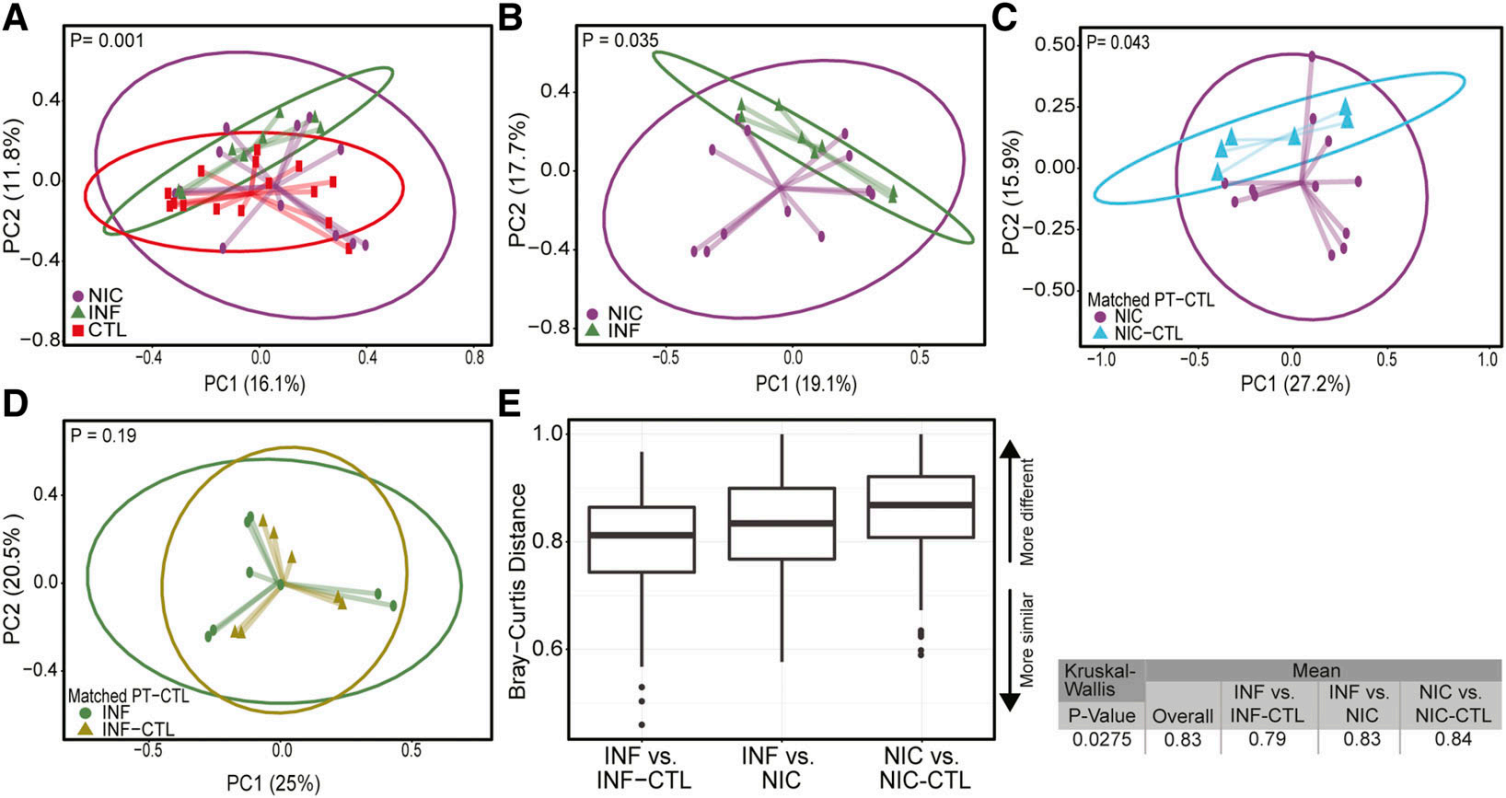
**NIC- and INF-CVID exhibit dissimilar gut microbiome compositions. (A–D)** Beta diversity between (A) NIC-CVID (*n* = 6), INF-CVID (*n* = 5), and household CTL (*n* = 8); (B) NIC- and INF-CVID; (C) NIC-CVID and their own household CTL; and (D) INF-CVID and their own household CTL. **(E)** Inter-group dissimilarities in gut microbiota composition between CVID patients and household CTL showed that the NIC-CVID group has a greater variation in gut microbiota than other groups (P = 0.027). Principal coordinate analysis plots of Bray–Curtis distances of fecal microbiota generated from mWGS sequence analysis. PC1, principal coordinate 1; PC2, principal coordinate 2. Analysis used weighted Bray–Curtis in A and B, unweighted Bray–Curtis in C and D, and the Kruskal–Wallis test in E. In E, only the statistically significant dissimilarities were included. Closer to 1 means the samples are more dissimilar. Total of 6 NIC, 4 NIC-CTL, 5 INF, and 4 INF-CTL were included.

Beta diversity captures differences in microbiota composition between two groups (25). To identify potential associations between gut microbial composition and CVID phenotype, we used the Bray‒Curtis dissimilarity matrix to cluster the metagenome using Agile Toolkit for Incisive Microbial Analysis (ATIMA), developed by the Center for Metagenomics and Microbiome Research at the Baylor College of Medicine (BCM) (26, 27). CVID patients’ bacterial microbiomes clustered separately from household CTLs and INF-CVID patients (Fig. 2, A and B).

Next, we compared each CVID phenotype with their household CTLs. The microbial composition of NIC-CVID patients was distinct from that of their household CTLs (Fig. 2 C), whereas the microbiota composition of INF-CVID patients did not significantly differ from that of their household CTLs (Fig. 2 D).

We next compared inter-group dissimilarities in gut microbiota composition. We found the NIC-CVID group had greater microbiota variation from their household CTLs compared with the other groups (Fig. 2 E). These findings indicate that NIC-CVID is associated with a significant shift in gut microbiome composition that overcomes the similarities that can be shared due to kinship and diet (28, 29, 30).

### Distinct microbial species are associated with NIC-CVID and INF-CVID patients

**Figure 3.**
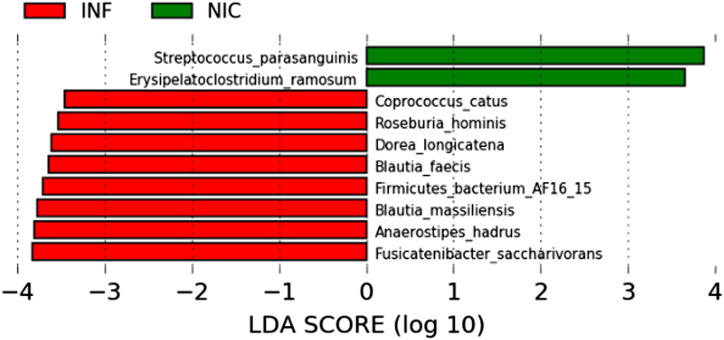
**LDA of differentially abundant species in fecal microbiota between NIC- and INF-CVID.** This analysis revealed significant differences in the gut microbiome of NIC-CVID compared with INF-CVID. LDA scores (log_10_) > 2 and P value <0.05 are shown.

We used linear discriminant analysis (LDA) and LDA effect size (LEfSe) to identify microbes differentially associated with NIC-CVID or INF-CVID (31). LEfSe couples standard tests for statistical significance with additional tests encoding biological consistency and effect relevance to determine the features, such as organisms, clades, operational taxonomic units, genes, or functions, most likely to explain differences between classes (31). We found significant differences in the gut microbiome composition of NIC-CVID and INF-CVID patients at the species level (Fig. 3). The discriminant species for the NIC-CVID group were *Streptococcus parasanguinis* and *Erysipelatoclostridium ramosum*. Both are pathobionts reported to cause severe infections in immunocompromised hosts (32, 33). In contrast, the microbiome of INF-CVID patients showed a preponderance of several microbes associated with anti-inflammatory effects, including *Fusicatenibacter saccharivorans*, *Dorea longicatena*, and *Blautia faecis* (34, 35, 36). Additionally, we identified in the gut microbiome of INF-CVID patients an enrichment of microbes that are associated with healthy metabolism, including *Anaerostipes hadrus* (37), *Coprococcus catus* (38), and *Roseburia hominis* (39).

### A new CVID-FMT gut dysbiosis model in GF mice

**Figure 4.**
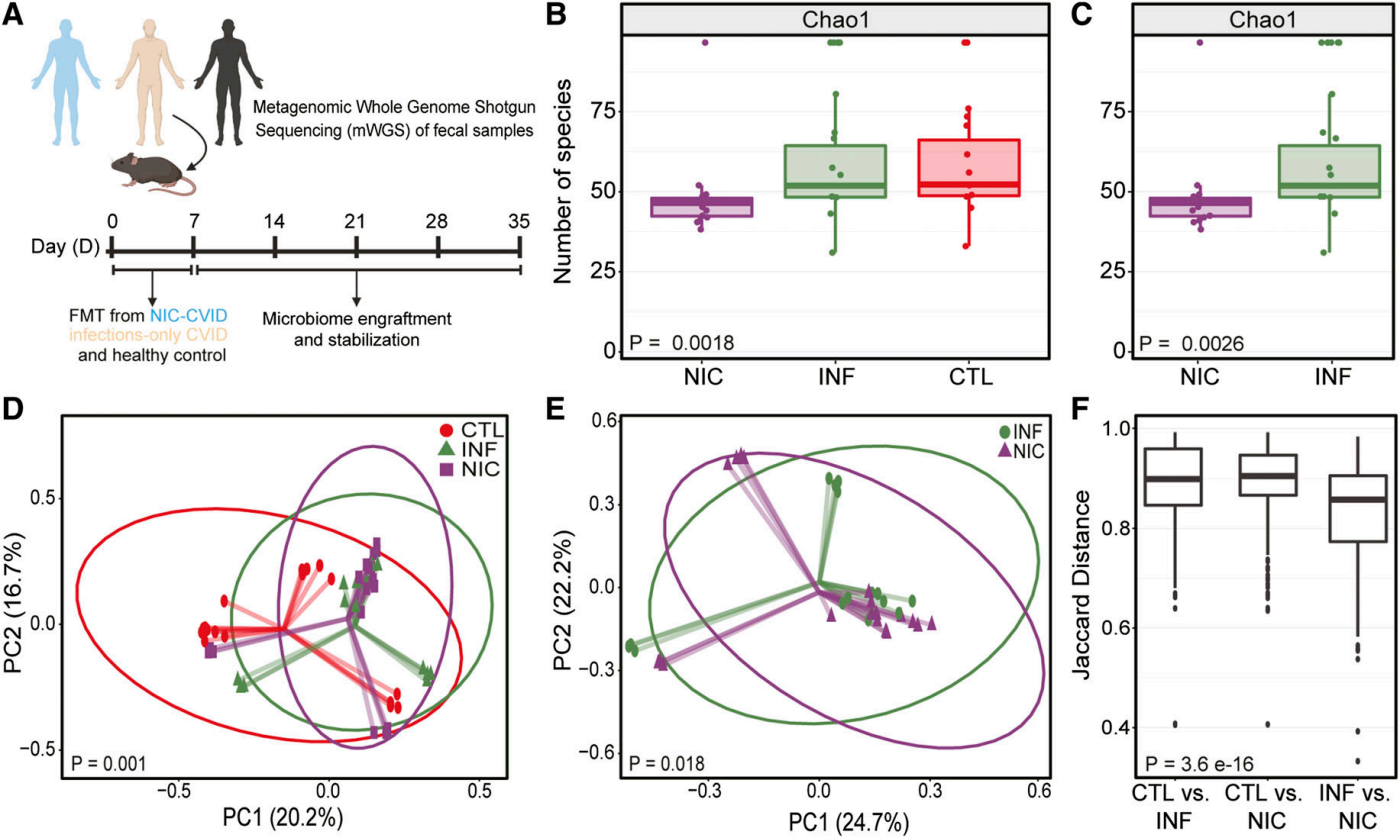
**FMT from CVID patients to GF mice recapitulates CVID patients’ gut dysbiosis. (A)** Experimental design of FMT from NIC- and INF-CVID patients and household CTL to GF (C57BL/6J) mice. Mice were orally gavaged (two to three times for 1 wk at 200 μl/dose) with fecal matter. Mice were allowed 30 days for the microbiome to stabilize. Sampling was performed at day 30 (D30) following FMT from NIC-CVID, INF-CVID, and household CTL donors. **(B)** Alpha diversity between NIC, INF, and CTL FMT recipients. **(C)** Alpha diversity between NIC- and INF-FMT recipients. Beta diversity between NIC-FMT, INF-FMT, and CTL-FMT. **(D)** Beta diversity between NIC-FMT, INF-FMT, and CTL-FMT recipients. **(E)** Beta Diversity between NIC-FMT and INF-FMT recipients only. Axis labels indicate the percentage of variance explained by respective principal coordinate axis. PC1, principal coordinate 1; PC2, principal coordinate 2. Donors (NIC-CVID *n* = 3, INF-CVID *n* = 3, and CTL *n* = 3). GF mice aged 8–12 wk. NIC-FMT *n* = 19 (16 males and 3 females), INF-FMT *n* = 16 (12 males and 4 females), and CTL-FMT *n* = 17 (13 males and 4 females). The analysis was performed by Kruskal–Wallis H test in B and C and by the Mann–Whitney *U* test in D and E. **(F)** Inter-group dissimilarities in gut microbiome composition between FMT recipients were most notable between FMT recipients from patients with CVID and their household CTLs FMT recipients; the closer to 1 means the samples are more dissimilar. Analysis was performed using the Kruskal–Wallis test. Panel A was created using https://BioRender.com.

Although CVID is considered the most common treatable inborn error of immunity in adults, it is still a rare and heterogeneous disease. A broader understanding of the role of the gut microbiome and its impact on immune regulation in CVID patients remains unclear. To determine the degree to which FMT would recapitulate differences in microbial composition observed in our human participants, we compared microbial communities between fecal matter from CVID patients, household CTLs, and FMT-recipient mice (Fig. 4 A).

**Figure S1.**
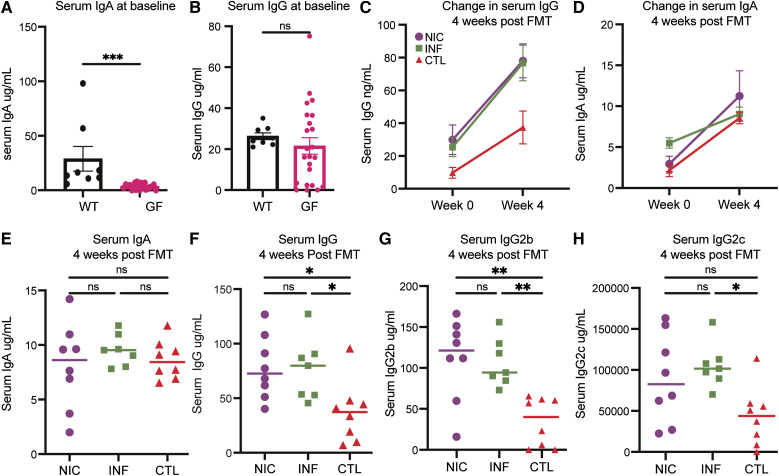
**Ig levels in GF mice at baseline and changes after FMT. (A–D)** GF mice have lower serum IgA compared with (A) WT and (B) variable IgG levels. Serum IgG (C) and serum IgA (D) increased in all recipients 30 days following FMT. **(E and F)** Total serum IgG (E) and IgG2b (F) were higher in NIC-FMT and INF-FMT recipients compared with CTLs. IgG2c (F) was higher than CTL-FT. All male mice aged 8–12 wk. Measured by ELISA. WT (C57BL/6J *n* = 8), GF (NIC-FMT *n* = 8, INF-FMT *n* = 7, and CTL-FMT *n* = 8). Unpaired *t* test for A and B and one-way ANOVA for E–H. *P < 0.05. WT, wild-type.

**Figure S2.**
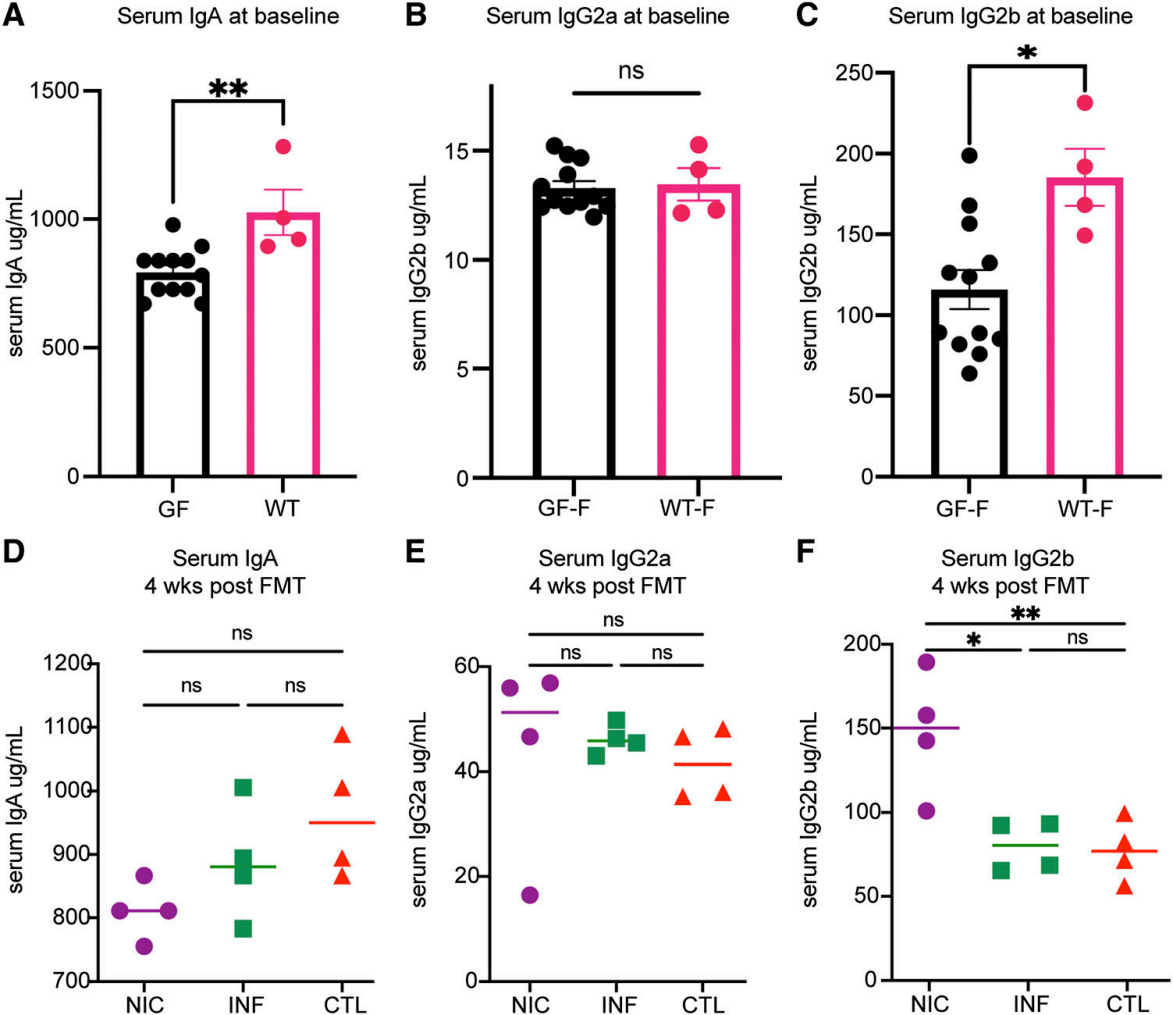
**Serum Ig levels before and after FMT in female mice.** Serum IgA, IgG2a, and IgG2b levels were measured in female mice at baseline before FMT and 4 wk after FMT. **(A)** Baseline IgA levels in GF and wild-type (WT) mice, with significant differences indicated by **. **(B)** Baseline IgG2a levels in GF and WT mice, with no significant difference observed (ns). **(C)** Baseline IgG2b levels, with significant differences indicated by *. Serum levels of IgA, IgG2a, and IgG2b were also measured at 4 wk after FMT for mice receiving FMT from NIC, INF, and CTL groups. **(D)** IgA levels at 4 wk after FMT, with no significant differences observed (ns). **(E)** IgG2a levels at 4 wk post-FMT, with no significant differences (ns). **(F)** IgG2b levels, with significant differences indicated by * and **.

GF mice have low serum and fecal IgA and underdeveloped Peyer patches, as well as small and underdeveloped mesenteric lymph nodes (40). In addition, introducing normal flora into GF mice restores their capacity to produce mucosal and systemic immune responses (41). Consistent with these findings, our pilot studies showed that GF(C57BL/6J) mice had undetectable serum IgA, variable serum IgG, and low fecal IgA/IgG levels (0–10 µg/ml and 0–3 ng/ml, respectively) at baseline (Fig. S1, A and B). 4 wk following FMT, serum IgA levels increased in all FMT recipients (Fig. S1 C). In addition, serum IgG increased (Fig. S1 D), whereas fecal IgG levels remained low (0–6 ng/ml, similar to fecal IgG levels in WT C57BL/6J mice) housed in a specific-pathogen-free facility. We noted interesting differences when we compared the Ig levels between FMT groups. First, there was no significant difference in serum IgA among FMT recipients following FMT (Fig. S1 E). In contrast, total serum IgG was higher in both NIC-FMT and INF-FMT recipients compared with CTL-FMT recipients (Fig. S1 F). Notably, the increase in IgG subclasses differed per FMT group. IgG2b was significantly higher in both NIC-FMT and INF-FMT recipients compared with CTL-FMT (Fig. S1 G), while IgG2c was higher in INF-CTL compared with all other groups (Fig. S1 H). We measured IgG2c instead of IgG2a because C57BL/6J mice produce this isotype in place of IgG2a (42). To assess whether similar trends were observed in female mice, we analyzed IgG subclass levels in female FMT recipients and found consistent patterns, as shown in Fig. S2.

IgG2c in mice is produced as a result of Th1 response and INFγ production (43, 44), while IgG2b binds to FcγRIII and IV, activating FcγRs, which has been shown to induce autoimmunity, such as arthritis (45) and thrombocytopenia (46). The antibody responses in CVID-FMT recipients suggest a potential inflammatory reaction to FMT compared with CTL-FMT recipients. However, these findings require further validation.

**Figure S3.**
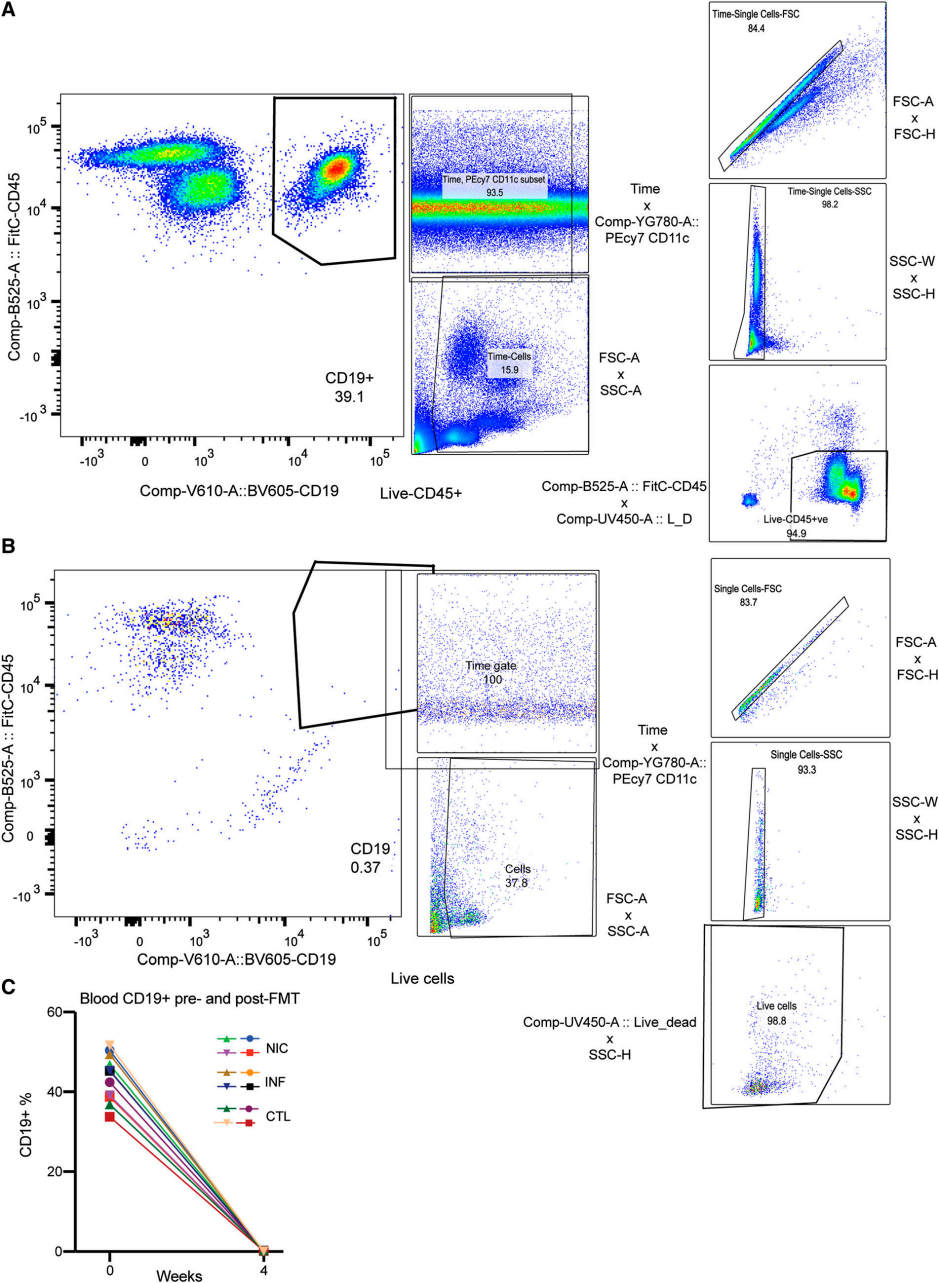
**Flow Cytometry for mice whole blood before and after B-cell depletion. (A and B)** Gating strategy and flow cytometry data on whole blood from the same mouse at baseline **(A)** and 6 wk following anti-CD20 treatment (B). **(C)** B cells in whole blood from mice treated with anti-CD20. Representative of one experiment, *n* = 4 mice/group. All male mice, ages 8–12 wk.

**Figure S4.**
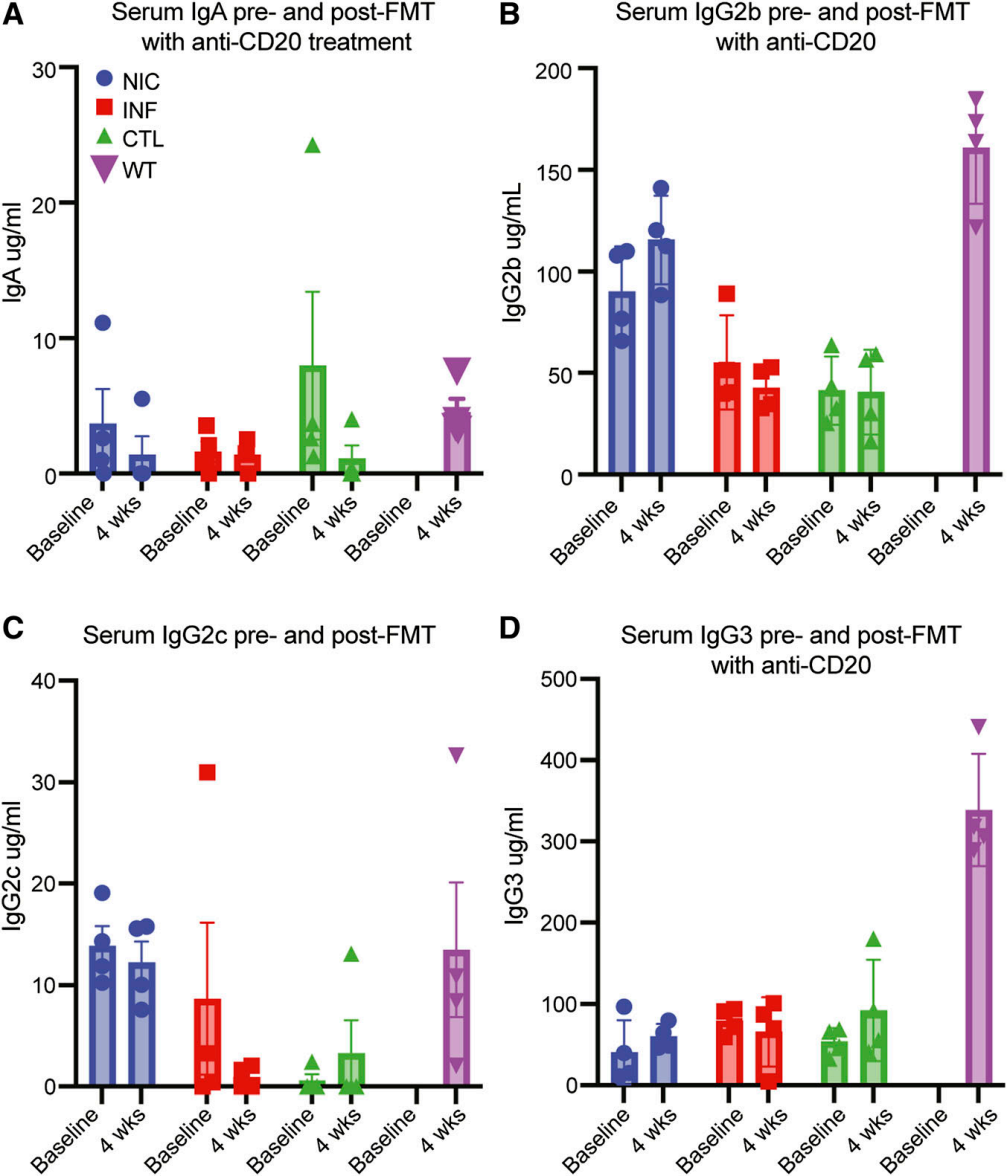
**Ig levels in FMT-recipient mice following anti-CD20 treatment. (A–D)** (A) IgA levels, (B) IgG2b levels, (C) IgG2c, and (D) IgG3 remained low after treatment with anti-CD20. Representation of one experiment, *n* = 4 mice/group. All female mice, ages 8–12 wk. Sera from wild-type (WT) (C57BL/6J) female mice housed in a specific-pathogen-free facility at BCM were used for comparison.

To prevent the development of anti-commensal antibody responses in FMT recipients (47), we pretreated GF mice with 100–250 µg anti-mouse CD20 monoclonal antibody (mAb) intraperitoneally every 2 wk to prevent the development of anti-commensal antibody responses in FMT recipients (47). Fig. S3, A and B show our flow cytometry gating strategy to assess mouse blood for B cells before and after anti-CD20 depletion. Fig. S4 C shows successful B cell depletion following anti-CD20 treatment. With this approach, we induced relative hypogammaglobulinemia (Fig. S3, A–D). The rationale for B cell depletion is to prevent the production of specific antibodies to new antigens (48), generating a humoral immune defect that resembles CVID. No significant differences in FMT engraftment or mouse health were noted in mice treated with anti-CD20 mAb.

### FMT from CVID patients to GF mice recapitulates CVID patients’ gut dysbiosis

We examined broad community metrics, including alpha and beta diversity, to characterize the overall similarity between donor and recipient communities. FMT donors were selected based on clinical phenotype (NIC-CVID, INF-CVID, or household CTL) and stool sample availability. This ensured representation from each group to assess whether microbiota differences between NIC-CVID and INF-CVID patients persisted after FMT into GF mice. Different groups of mice received microbiota from different donors (three donors of the same clinical phenotype per FMT recipient group).

4 wk following FMT, there was a significant difference in microbial richness and alpha diversity between NIC-FMT, INF-FMT, and CTL-FMT recipients (Fig. 4 B). We also found a significant difference in gut microbial richness and alpha diversity between NIC-FMT and INF-FMT recipients (Fig. 4 C). In addition, beta diversity measurements using unweighted and weighted UniFrac distances revealed that the gut microbiome composition was significantly different between the three FMT groups (Fig. 4 D). Notably, the microbiota composition of NIC-FMT recipients was distinct from INF-FMT recipients (Fig. 4 E). In addition, inter-group analysis in gut microbiota composition identified dissimilarities between FMT recipients, most notably between CVID-FMT and CTL-FMT recipients (Fig. 4 F). Taken together, these results demonstrate that FMT mouse recipients predominantly exhibited gut microbiome compositional aberrations resembling what was seen in CVID donors.

**Figure 5.**
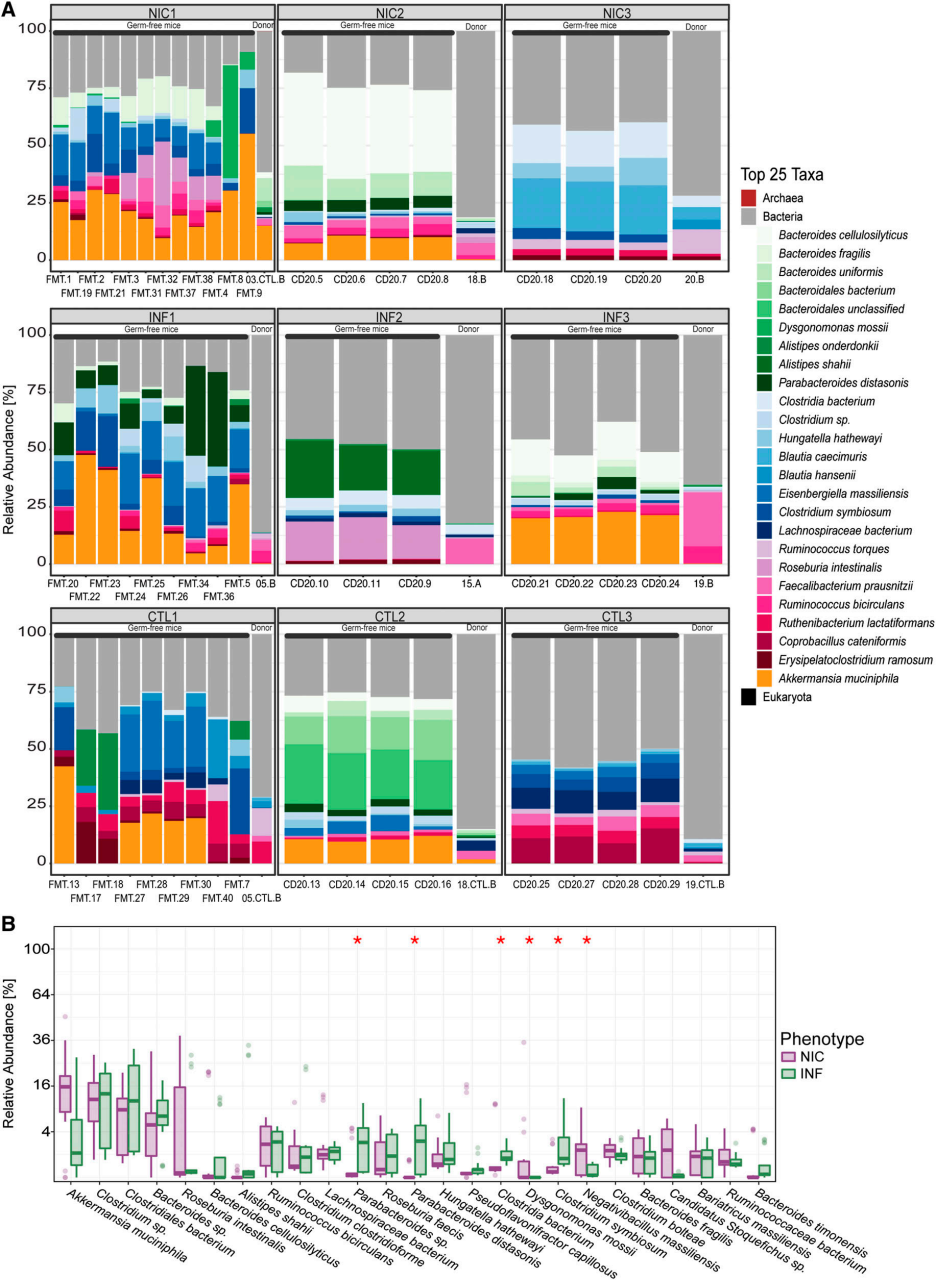
**Relative abundance plots comparing human microbiome from donors to FMT-recipient mice (top 25 taxa). (A)** Comparison between each human donor and mouse 4 wk following FMT indicates no statistically significant difference in the relative abundance between human donors and the mouse FMT recipient. Each FMT is represented here, including the human donor (NIC, INF, or CTL) and the recipient FMT group. A is a representation of the top 25 taxa. **(B)** Comparison between NIC-FMT and INF-FMT microbes’ relative abundance 4 wk following FMT. The graph is representative of the top 25 taxa. The top 25 most abundant taxa were selected to ensure consistent and meaningful comparisons while minimizing potential artifacts from low abundance. Significant P value <0.05 (marked by an asterisk).

We compared the relative abundance of the top 25 most abundant taxa between human fecal donors and FMT recipients (Fig. 5 A). FMT recipients retained key microbial taxa from their respective human donors, including *Bacteroides* sp., *Clostridium* sp., and *Akkermansia muciniphila*, suggesting successful microbiota transfer. However, some taxa, such as *Klebsiella* sp., were present in low abundance in one NIC-CVID donor but were not detected in recipient mice, indicating that host-specific factors influence microbial engraftment. Given the complexity of gut microbiota composition and the inherent variability in cross-species microbiota transfer, we focused our analysis on the top 25 most abundant taxa to ensure robust comparisons while minimizing potential artifacts from low-abundance taxa.

Finally, we examined species-level differences between NIC-FMT and INF-FMT recipients. A representation of the mice’s fecal microbiome that compares the relative abundance of the top 25 most abundant taxa between NIC-FMT and INF-FMT recipients is shown in (Fig. 5 B). Similar to what we observed in CVID patients, NIC-FMT recipients had a higher relative abundance of microbes that can potentially cause opportunistic infections in immunocompromised individuals, including *Dysgonomonas mossii* and *Negativebacillus massiliensis. D. mossii* is a Gram-negative, anaerobic, coccobacillus-shaped bacterium within the phylum Bacteroidetes that has been reported to cause opportunistic infections in patients with type 1 diabetes and cancer (49, 50, 51). Similarly, *N. massiliensis* is a rare microbe that caused meningitis in a patient with Whipple syndrome (52). On the other hand, INF-FMT recipients had a higher relative abundance of potentially beneficial microbes, including *Clostridium symbiosum* and *Parabacteroides distasonis*. *C. symbiosum* is a short-chain fatty acid producer associated with immunomodulatory and anti-inflammatory effects (53). Adding *C. symbiosum* to the microbiota of a malnutrition mouse model ameliorated growth and metabolic abnormalities in the recipient mice (54). *P. distasonis* is one of 18 core members in the human gut microbiota (55) and thought to have critical physiological functions in its hosts. *P. distasonis* produces succinate (which activates gut glucogenesis) and transforms primary bile acids into secondary bile acids (56). Both succinate and secondary bile acids can promote gut barrier integrity and reduce inflammation in the gut of obese mice (57).

Taken together, our mWGS analysis of fecal matter from CVID patients and FMT-recipient mice revealed a high level of similarity between humans and mice, both in diversity metrics and in potential function. Both NIC-CVID patients and NIC-FMT recipients harbored potential pathogenic microbes associated with opportunistic infections in immunocompromised hosts, whereas INF-CVID patients and INF-FMT recipients harbored microbes with beneficial metabolic functions and potential anti-inflammatory capacity.

## Discussion

In the present study, we performed mWGS on the gut microbiomes from NIC-CVID and INF-CVID patients, as well as their healthy household CTLs. To overcome intraindividual microbial variations that can be missed when only a single sample collection is used, we collected two samples from each patient and household CTL for a more accurate assessment of the microbiome composition (58). Additionally, we included healthy household members as CTLs for diet and environmental factors (59). Household members share more of their gut microbes compared with unrelated individuals, and intimate partners share even more gut microbiota than other household members (28, 29). Using these robust methods, we were able to further characterize gut microbiome composition in CVID patients. We identified specific microbes that were more abundant in NIC-CVID patients, including *S. parasanguinis* and *E. ramosum. S. parasanguinis* is predominantly an oral cavity microbe that belongs to the viridans group streptococci. Although viridans group streptococci are generally considered to be of low pathogenic potential in immunocompetent individuals, they can cause invasive diseases such as endocarditis, intra-abdominal infection, and shock (60). *S. parasanguinis* is known to produce hydrogen peroxide (61) and has been reported to cause invasive infections, such as infective endocarditis and pneumonia, in immunocompromised hosts (32, 62). Additionally, the presence of *S. parasanguinis* in the gut is associated with dysbiosis in inflammatory bowel disease patients, owing to oxidative stress resistance in such bacteria (63). Hence, it is plausible that *S. parasanguinis* contributes to gut dysbiosis and immune dysregulation in NIC-CVID. We also found that *E. ramosum* is more abundant in the gut microbiome of NIC-CVID patients. *E. ramosum* belongs to the clostridia group and has been shown to cause severe infections, particularly in immunocompromised patients (33). Interestingly, *E. ramosum* produces an IgA protease that is capable of cleaving human IgA (64). *E. ramosum* has been shown recently to be over 80-fold enriched in individuals with selective IgA deficiency, especially in those with autoreactive anti-IgA antibodies, suggesting a potential role for this pathobiont as an autoimmune trigger (65).

In INF-CVID patients, we noted an increased abundance of several microbes associated with potential anti-inflammatory effects, including *F. saccharivorans*, *D. longicatena*, and *B. faecis*. We also identified microbes associated with healthy metabolism, including *A. hadrus*, *C. catus*, *R. hominis*, *Blautia massiliensis*, and *Firmicutes bacterium.*

The most abundant bacteria in INF-CVID patients was *F. saccharivorans*, a species of the Clostridia class. Its abundance is associated with ulcerative colitis remission (34). In contrast, its decrease is associated with increased ulcerative colitis disease activity, which has been attributed to its immunomodulatory effects and its ability to induce IL-10 production in humans and mice (34, 66). Similarly, the presence of *D. longicatena* in the gut microbiome is associated with Crohn’s disease remission (35). The second most abundant bacteria in the gut of INF-CVID patients was *A. hadrus*, a human-derived butyrate-producing species. In contrast, *A. hardus* was shown in mice to be beneficial by increasing butyrate levels in the gut and harmful by potentially causing worse chemically induced colitis (37). Butyrate is produced when gut microbes ferment dietary fiber and is considered a health-promoting molecule due to its anti-inflammatory (67) and antineoplastic potential (68). We also revealed that two of the *Blautia* species were enriched in the INF-CVID gut microbiome. *Blautia* sp. can metabolize polymethoxyflavones, which are major bioactive flavonoids with various biological activities, including anti-inflammatory and anticancer effects (36, 69). Finally, we observed *Firmicutes* was enriched in INF-CVID patients. Two studies that used 16S rRNA gene sequencing for CVID gut microbiomes identified an increase in some *Firmicutes* in CVID (14, 70) metabolizing polymethoxyflavones, which are major bioactive flavonoids with various biological activities, including anti-inflammatory and anticancer effects, producing butyrate, and supporting healthy metabolism (71). Notably, *Firmicutes* harbors H_2_-oxidizing properties that promote more efficient energy extraction from food (72). Although an abundance of *Firmicutes* in the gut microbiome is associated with obesity (71, 73), this property of *Firmicutes* might be beneficial in CVID patients, as many with enteropathy develop malnutrition (74). Overall, the gut microbiome in INF-CVID patients was enriched with microbes that have been associated with a healthy metabolism and anti-inflammatory effects. In contrast, the NIC-CVID microbiome was enriched with inflammation-associated microbes, especially in the immunocompromised host.

In addition to our comprehensive characterization of the CVID gut microbiome, we provided a proof of concept that FMT from CVID patients to GF mice recapitulates microbiome alterations seen in CVID patients. The primary goal of this model was to evaluate whether gut microbiota differences observed in NIC-CVID and INF-CVID patients could be transferred and maintained in mice. While this approach does not fully recapitulate the immune dysfunction seen in CVID, it provides a controlled system to assess microbiota-driven effects in the absence of host immune confounders. Our findings demonstrate that FMT recipients retained key microbial shifts observed in human donors, supporting the role of microbiota alterations in NIC-CVID–associated immune dysregulation.

As far as we are aware, our model is the first to use B cell depletion to induce hypogammaglobulinemia and prevent the generation of specific antibody responses against transplanted human microbiota, creating an antibody defect that resembles the CVID immunophenotype. While anti-CD20 mAb is commonly used in CVID patients with immune dysregulatory conditions such as immune-mediated thrombocytopenia and granulomatous lymphocytic interstitial lung disease, the immunosuppressive effects of anti-CD20 should be considered when interpreting the results. It is important to note, however, that while the model serves as a controlled means to study microbiota-driven immune dysregulation, it does not fully replicate the immune dysfunction seen in human CVID. Even though the highest abundance of microbes in mice was not the same as in CVID patients, we noted the same potential pathogenicity and function in the gut microbiome of both mice and humans. The absence of marked shifts after B cell depletion further emphasizes the complexity of microbial–immune interactions and highlights the distinctions between human and murine immune responses. The relative abundance of microbes associated with opportunistic infections and potential pro-inflammatory capacities was enriched in NIC-CVID patients, while microbes associated with a healthy metabolism and potential anti-inflammatory capacities were enriched in INF-CVID patients and INF-FMT recipients. In future studies, we believe this model may allow us to assess the impact of microbiome manipulation on immune responses and test therapeutics to ameliorate microbiome-associated immune dysregulation in CVID patients.

Although we did not detect a significant difference in alpha diversity between CVID patients and household CTLs or between NIC-CVID and INF-CVID, we noted that alpha diversity in the NIC-CVID participants was qualitatively lower compared with INF-CVID and household CTLs. Previous studies showed that alpha diversity was lower in CVID patients compared with a general population healthy CTL and household CTLs using 16S rRNA gene sequencing (14, 20). However, smaller studies using mWGS showed that CVID patients (with no significant complications) had increased bacterial diversity compared with their household CTLs (75). Unlike 16S rRNA sequencing, mWGS reads all genomic DNA in a sample, rather than just one specific region of DNA, which allows the identification and profiling of all microbial genes present in the sample (the metagenome). Thus, metagenomic profiling often provides species-level assignment (76).

Our study has some limitations. Owing to the rare nature of inborn errors of immunity, this study comprised a small sample size. Additionally, our strict exclusion criteria eliminated patients with acute illness, infection, or recent use of antimicrobial agents. However, our longitudinal design and the inclusion of household CTLs helped mitigate these limitations by accounting for shared diets and environments (24, 29). To further strengthen our findings, we collected two stool samples per subject to reduce intraindividual variability and improve microbiome assessment (58).

Due to the small sample size, our study was not powered to formally assess the contribution of demographic factors such as age and sex to microbiome composition differences. However, since all participants were adults and both sexes were represented in each group, these factors are unlikely to be the primary drivers of the observed differences. Despite these limitations, our study provides a proof of concept that gut dysbiosis in NIC-CVID can be recapitulated in GF mice, supporting its role in immune dysregulation.

Our goal for this study was not to generate a CVID mouse model but rather to create a gut dysbiosis model that could potentially be used to further model mucosal immune dysregulation in CVID. In addition, the model developed in this study may allow us to assess the impact of microbiome manipulation on immune responses and test therapeutics to ameliorate immune dysregulation in an immunocompromised host.

In conclusion, our findings highlight distinct gut microbiome shifts in CVID patients. The microbiota of INF-CVID patients appears less pathogenic and exhibits greater anti-inflammatory potential compared with NIC-CVID.

## Materials and methods

### Recruitment of CVID patients

Patients were diagnosed with CVID by their treating clinical immunologists. One patient initially presented with a CVID phenotype and was enrolled based on clinical findings. A RAG1 mutation was identified after enrollment, sample collection, and analysis. Another patient had lymphoma as the first presentation but exhibited a phenotype consistent with CVID, including low memory B cells and no immune recovery after treatment, making CVID the more likely diagnosis as determined by the treating physician. Table 1 summarizes patients’ characteristics, and Table S1 summarizes the basic demographic of the household CTLs.

**Table 1.** Patients’ demographics and summary of clinical and immunological phenotype

Subject	CVID phenotype	Sex	Race/ethnicity	Age at diagnosis	Age at enrollment	Treatment	Active NIC at time of sample collection	History of immune suppression used	# years from immune suppression use to fecal sampling	Baseline IgA	Baseline IgM	Total B cells	CD27^+^ CD21low	CD3^+^	CD4^+^	CD8^+^
1	INF	M	White	28	30	IVIG	N/A	N/A	N/A	<15	<5	6% (430)	9.5% (41)	75% (1,697)	31.9% (722)	40.3% (912)
3	INF	M	White	50	64	IVIG	N/A	Mesalamine	2 mo	<5	<5	0	0	95% (1,949)	27.9% (572)	74% (1,518)
5	INF^a^	M	White	43	50	IVIG	N/A	Rituximab + hyper CVD	20 years	<10	11	7.2% (98)	2.1% (1)	70% (1,084)	32.7% (445)	40.1 (545)
15	INF	F	White	52	57	SCIG	N/A	N/A	N/A	<5	8.5	18.2 (367)	21.5% (79)	77% (1,461)	44.3% (841)	31.7 (602)
19	INF	F	White		57	HyQvia	N/A	N/A	N/A	N/A	N/A	N/A	N/A	N/A	N/A	N/A
2	NIC	F	White	41	70	IVIG	Sjogren syndrome, Barrett's esophagus, B12 deficiency, and chronic diarrhea	N/A	N/A	<6	11.8	9.9% (198)	3.9% (8)	63% (1,106)	29% (509)	32% (562)
4	NIC	F	White/Hispanic	32	42	HyQvia	CVID-entropathy^c^	Budesonide	On and off 3 years	<5	<5	25.4% (283)	1% (4)	69% (770)	42% (469)	25.8% (288)
11	NIC	M	White	33	52	SCIG	GLILD, liver NRH, CVID entropathy, and splenomegaly	Ustekinumab and oral IgG	3 mo	<6	7.3	15% (113)	3.1% (4)	73 (471)	40% (258)	27% (174)
12	NIC^b^	F	White/Hispanic	34	38	IVIG	Alopecia, vitiligo, premature ovarian failure, and cytopenia	None	N/A	76	76.3	3	N/A	80	47	31
18	NIC	F	White	59	64	IVIG	GLILD, NRH, and thrombocytopenia	Rituximab	2 years	<9	339	21% (254)	N/A	70% (892)	53% (627)	14% (168)
20	NIC	M	White		21	HyQvia	Cerebral vasculitis and granulomatous encephalitis	N/A	N/A	N/A	N/A	N/A	N/A	N/A	N/A	N/A

GLILD: granulomatous lymphocytic interstitial lung disease. NRH: nodular regenerative hyperplasia. Hyper CVAD includes cyclophosphamide, vincristine, Adriamycin (doxorubicin), dexamethasone, methotrexate, and cytarabine. N/A: Data were not available for those patients.

a Patient developed EBV-related lymphoma 20 years ago (currently in remission), treated with rituximab, never recovered Igs, has 0 switched memory B cells, undetectable IgA, and IgM.

b Patient presented with CVID phenotype at age 34, later found to have RAG1 mutation.

c CVID enteropathy includes inflammatory bowel disease like enteritis.

We excluded patients with an acute infection/illness and those who received antibiotics for an acute infection 30 days before enrollment. We defined NIC-CVID patients as having severe forms of autoimmunity/immune dysregulation associated with CVID (1) (i.e., granulomatous interstitial lung disease, colitis, nodular regenerative hyperplasia, lymphoproliferation, and severe cytopenia). Common autoimmunity, such as hypothyroidism alone, was not considered NIC. CVID enteropathy was classified based on the presence of chronic gastrointestinal symptoms. Acute gastrointestinal infections were ruled out through appropriate testing, and when clinically indicated, endoscopic evaluation was performed to assess for inflammation or structural abnormalities.

### Fecal matter collection for human subjects

Patients 1, 2, and 3 collected their stool samples using OMNIgene•GUT OM-200. However, due to the need for FMT, all other subjects collected fecal material at home and were instructed to immediately freeze the material. For sequencing, we used the same sequencing methodology, same primers, and same data analysis pipeline for all samples. Samples were kept frozen and were brought in or shipped on ice. Upon receipt, we inspected all samples to ensure they remained frozen and stored them at −80°C. Each subject and CTL provided two samples that were 20 days apart, and both samples were sequenced separately for metagenomic analysis. No pooling of samples was performed.

### Animal housing and handling

All experimental animal procedures were approved by the Institutional Animal Care and Use Committee at BCM. GF (*C57BL/6J*) mice were bred and maintained at the BCM Gnotobiotic Core in isolators or for the anti-CD20 mAb treatment in Tecniplast IsoP hermetically sealed positive-pressure individually ventilated cages. Following FMT, all mice were housed in sterile micro-isolator cages and fed ad libitum on standard chow. We used a total of 41 males and 11 females; skewing of the sex to males was due to availability in Gnotobiotic Core.

### Blood collection

Blood was collected from mice via facial cheek bleed using a lancet when alive and cardiac puncture after sacrifice.

### Ig measurements

Serum Igs were measured using ELISA kits (Thermo Fisher Scientific). All antibodies used in mice had no cross-reactivity with human antibodies.

### Flow cytometry

All flow cytometry procedures were performed on a BD LSRII (BD Biosciences) in collaboration with BCM’s Cytometry and Cell Sorting Core, and data were analyzed using FlowJo (Treestar). The following antibodies were purchased for flow cytometric analysis: CD45 FITC and CD19 BV605 (Thermo Fisher Scientific).

### B cell depletion using antibody treatment

In two separate experiments, we pretreated GF mice with anti-mouse CD20 mAb (clone 5D2, isotype IgG2a; Genentech) every 2 wk (47, 77). We procured this reagent through a material transfer agreement with Genentech. Mice received 100–250 µg treatments intraperitoneally weekly before FMT, followed by biweekly treatments for the duration of the experiments. B cell depletion created a state of hypogammaglobulinemia, not agammaglobulinemia, and blunted the ability of the mice to produce antibodies against microbes from the FMT, which led to a state of antibody deficiency that resembles the immunophenotype of CVID patients.

### Mouse fecal pellet collection

To evaluate the microbiome profile in GF mice, fresh fecal pellet samples from each mouse were collected in sterile 1.5-ml centrifuge tubes and stored at −80°C until DNA extraction.

### Fecal microbiome transplant

FMT experiments were performed as described previously (78). Fecal matter was thawed, diluted (100 mg/1 ml sterile PBS), passed through a 40-μm strainer thrice, and then frozen at −80°C. GF (*C57BL/6J*) mice (males and females, age 8–12 wk) were orally gavaged (two to three times over 1 wk at 200 μl/dose) with fecal matter from either NIC-CVID or INF-CVID patients or a healthy donor. Following FMT, mice were monitored for 30 days to allow microbiome stabilization. Blood and fecal samples were collected at baseline and 30 days later.

### DNA extraction from fecal samples

DNA was extracted from fecal pellets using the QIAamp 96 DNA QIAcube HT Kit (51551; Qiagen) with the following modifications. Before loading on the Qiacube robot, samples were incubated with 5 μl of lysozyme (10 mg/ml, L6876; Sigma-Aldrich), 1 μl of lysostaphin (5,000 U/ml, L9043; Sigma-Aldrich), and 1 μl mutanolysin (5,000 U/ml, M9901; Sigma-Aldrich) for a 30-min digest at 37°C. Next, samples were mechanically disrupted with 100-μl glass beads (0.1 diameters, 11079101; BioSpec Products) twice for 3 min each at 30 Hz (TissueLyser II; Qiagen) and then loaded onto the robot for extraction.

### Whole-genome shotgun sequencing

Libraries were prepared using the Nextera XT DNA Library Prep Kit (Illumina) according to the manufacturer’s instructions, except for using one-quarter of the recommended reaction volume. Whole-genome shotgun sequencing was predominantly carried out using a 2 × 150 bp (paired end) sequencing protocol on the NovaSeq 6000 Sequencing System (Illumina), according to the manufacturer’s manual. Sequencing was conducted at the Genome Technologies core facility at the Jackson Laboratory for Genomic Medicine, Farmington, CT, USA.

### Positive and negative CTLs

One sample of a defined, in-house mock community (25 diverse Gram-positive and Gram-negative bacteria and fungi) and a negative CTL (nuclease-free water; Qiagen) were included per extraction round. Additionally, a negative CTL of nuclease-free water was included for library generation, and one mock community sample was added to each sequencing run. A library/extraction negative CTL was sequenced if a library product was measurable on the Qubit 2.0 Fluorometer (Thermo Fisher Scientific) or identified on the 4200 TapeStation System (Agilent Technologies) using the High Sensitivity D1000 ScreenTape Assay (Agilent Technologies). The sequencing data were processed as described below.

### Data processing

Samples with <1.4 million reads were excluded, leaving 22 samples from CVID patients, 15 household CTLs, and 112 mouse fecal samples for analysis. Relative proportions were used for all analyses. All taxonomic features at the species level with a mean relative abundance of 0.01% (denoise function [79]) across the dataset were removed from the dataset to reduce potential false positives and allow for multiple hypothesis correction.

### Biomarker discovery with LEfSe

LEfSe pipeline (31) was used with default parameters (LDA score log [10] > 2.0) to identify discriminant taxa between sample groups. We opted to categorize our metagenomic profiles based on MetaPhlAn 4. MetaPhlAn 4 is a tool for profiling microbiome communities and uses a database of unique, clade-specific gene markers. It assigns fragments by mapping them against the gene markers database (80). MetaPhlAn is associated with higher accuracy and lower rates of false positivity (80, 81).

### mWGS data processing for taxonomic classification

mWGS comprehensively samples all genes in all organisms present in a given complex sample and identifies bacteria, viruses, and fungi. A host database consisting of GRCh38, Immuno Polymorphism Database (release 2.0.0, June 2018; build 60 [82]), NCBI UniVec clone vector sequences (build #10.0) (83), and Human GENCODE transcripts (release 25 [84]) was built for host-data removal. Trimmomatic (version 0.32 [85]) was used for Nextera adapter removal. All samples were subsampled using seqtk (https://github.com/lh3/seqtk) to the median read depth of 1,414,348.

The FASTQ files were processed with MetaPhlAn 4 (86) and the GATK pipeline Pathseq (v2.0. [87, 88]). PathSeq aligns the reads (trimmed, quality filtered [mean quality base score of 20], and deduplicated) that are a minimum 50-bp length at 95% identity to a reference of microbial genomes (viruses, bacteria, Eukaryota [including fungi], and archaea) from RefSeq (release 99, accessed: 3/2/2020 [89]). MetaPlan4 achieved the closest taxonomic classification to the expected values of our mock community and was subsequently used for this study.

### Statistical analysis

We used the R-based software ATIMA (27) to generate plots visualizing alpha diversity (richness and evenness), beta diversity (in-between sample differences), and taxa abundances (phylum-genus) through box plots, principal coordinate analysis ordinations, and heatmaps.

ATIMA enables rarefied and non-rarefied relative abundance analysis. We analyzed categorical variables using the nonparametric Mann–Whitney and Kruskal–Wallis tests for variables with two groups or ≥3 groups, respectively. P values were adjusted for multiple comparisons using the false discovery rate algorithm.

The inter-group dissimilarities (beta diversity) in gut microbiota composition were measured using the Bray–Curtis distance metrics. Bray–Curtis dissimilarity quantifies the differences in species populations between two different sites. The resulting number is between 0 and 1, with 0 denoting the highest similarity (two samples share the same species) and 1 denoting the highest dissimilarity.

To ensure statistical robustness and avoid pseudoreplication, we treated each mouse as an independent biological replicate, accounting for inter-mouse variability within experimental groups. Statistical comparisons were performed at the cohort level, ensuring that results reflect donor-dependent microbiome differences rather than within-group variation.

### Online supplemental material

Supplementary figures provide additional data supporting the Ig responses in GF mice following FMT (Figs. S1 and S2), flow cytometric validation of B cell depletion after anti-CD20 treatment (Fig. S3), and serum Ig levels after FMT in anti-CD20–treated mice (Fig. S4). Table S1 summarizes demographic and clinical data of household CTLs. These materials provide further validation and context for the murine modeling of CVID-associated microbiome alterations.

## Supplementary Material

Table S1 shows demographics of household CTLs.

## Data Availability

The datasets generated during and/or analyzed during the current study are available from the corresponding author on reasonable request.
