# Supplementary material for: Gut dysbiosis patterns in CVID patients with noninfectious complications observed in a germ-free mouse model through fecal microbiota transplantation
Source: J Hum Immun. 2025 Apr 23;1(1):e20250040. doi: 10.70962/jhi.20250040 (PMC12462835; doi:10.70962/jhi.20250040)
Supplement: Table S1 — shows demographics of household CTLs. [file jhi_20250040_table_s1.docx]

**Table S1** Demographics of household controls.

*Healthy control serving as a control for 2 CVID patients who were a mother and a son
