# Supplementary material for: Gut dysbiosis patterns in CVID patients with noninfectious complications observed in a germ-free mouse model through fecal microbiota transplantation
Source: J Hum Immun. Author manuscript; Available in PMC 2025 Sep 26. (PMC12462835; doi:10.70962/jhi.20250040)
Supplement: Supplementary file 1 [file NIHMS2108511-supplement-2.pdf]

JHI

Supplemental material

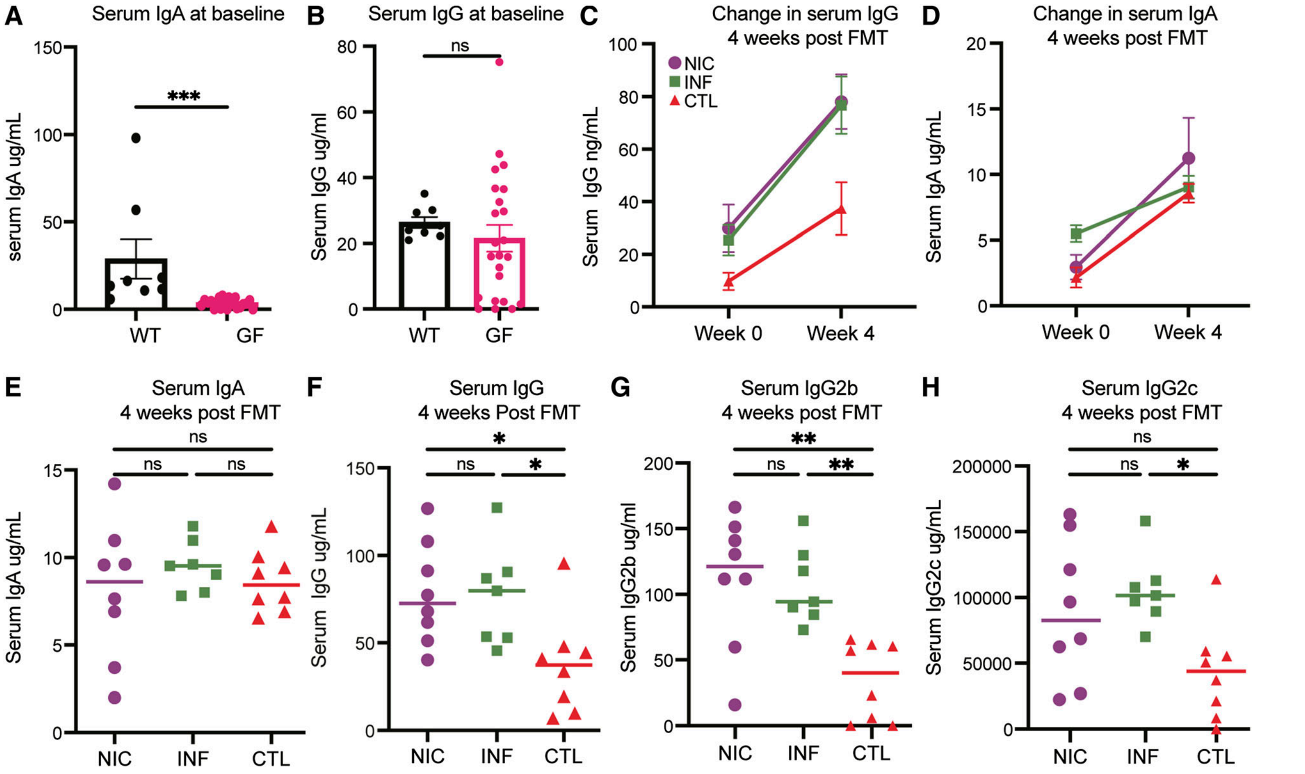

Figure S1. **Ig levels in GF mice at baseline and changes after FMT. (A–D)** GF mice have lower serum IgA compared with (A) WT and (B) variable IgG levels. Serum IgG (C) and serum IgA (D) increased in all recipients 30 days following FMT. **(E and F)** Total serum IgG (E) and IgG2b (F) were higher in NIC-FMT and INF-FMT recipients compared with CTLs. IgG2c (F) was higher than CTL-FT. All male mice aged 8–12 wk. Measured by ELISA. WT (C57BL/6J *n* = 8), GF (NIC-FMT *n* = 8, INF-FMT *n* = 7, and CTL-FMT *n* = 8). Unpaired *t* test for A and B and one-way ANOVA for E–H. *P < 0.05. WT, wild-type.

Downloaded from http://rupress.org/jhi/article-pdf/1/1/e20250040/1943074/jhi_20250040.pdf by Joud Hajjar on 01 September 2025

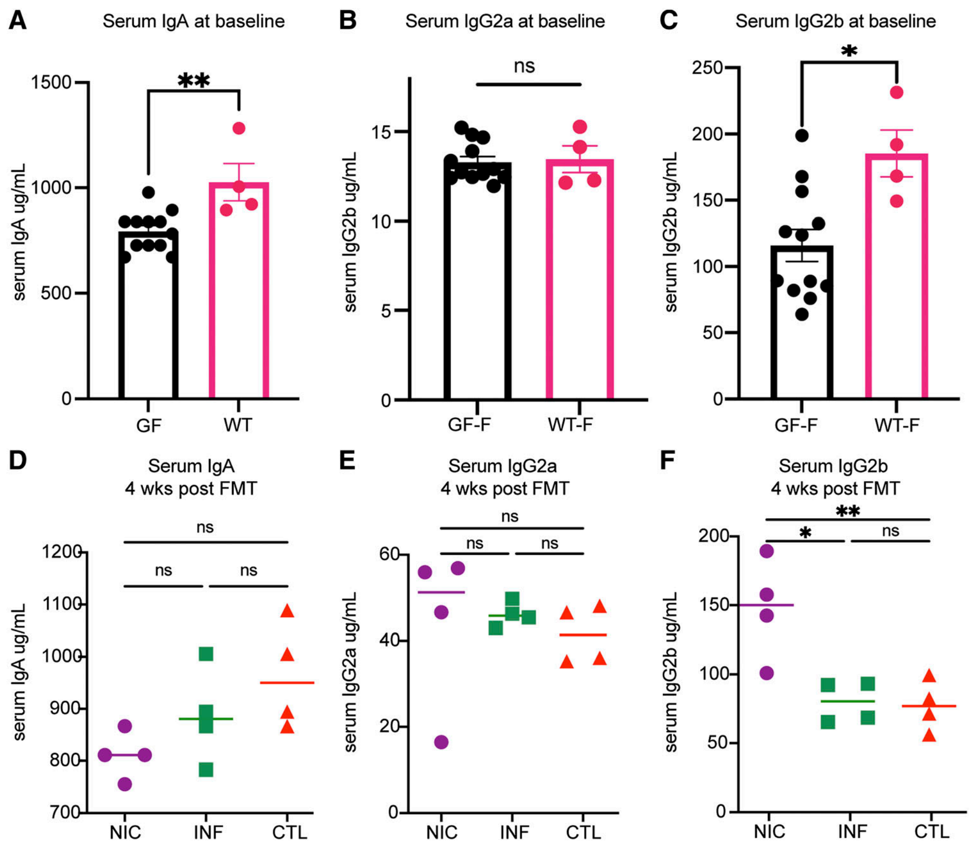

Figure S2. **Serum Ig levels before and after FMT in female mice.** Serum IgA, IgG2a, and IgG2b levels were measured in female mice at baseline before FMT and 4 wk after FMT. **(A)** Baseline IgA levels in GF and wild-type (WT) mice, with significant differences indicated by **. **(B)** Baseline IgG2a levels in GF and WT mice, with no significant difference observed (ns). **(C)** Baseline IgG2b levels, with significant differences indicated by *. Serum levels of IgA, IgG2a, and IgG2b were also measured at 4 wk after FMT for mice receiving FMT from NIC, INF, and CTL groups. **(D)** IgA levels at 4 wk after FMT, with no significant differences observed (ns). **(E)** IgG2a levels at 4 wk post-FMT, with no significant differences (ns). **(F)** IgG2b levels, with significant differences indicated by * and **.

Downloaded from http://rupress.org/jhi/article-pdf/1/1/e20250040/1943074/jhi_20250040.pdf by Joud Hajjar on 01 September 2025

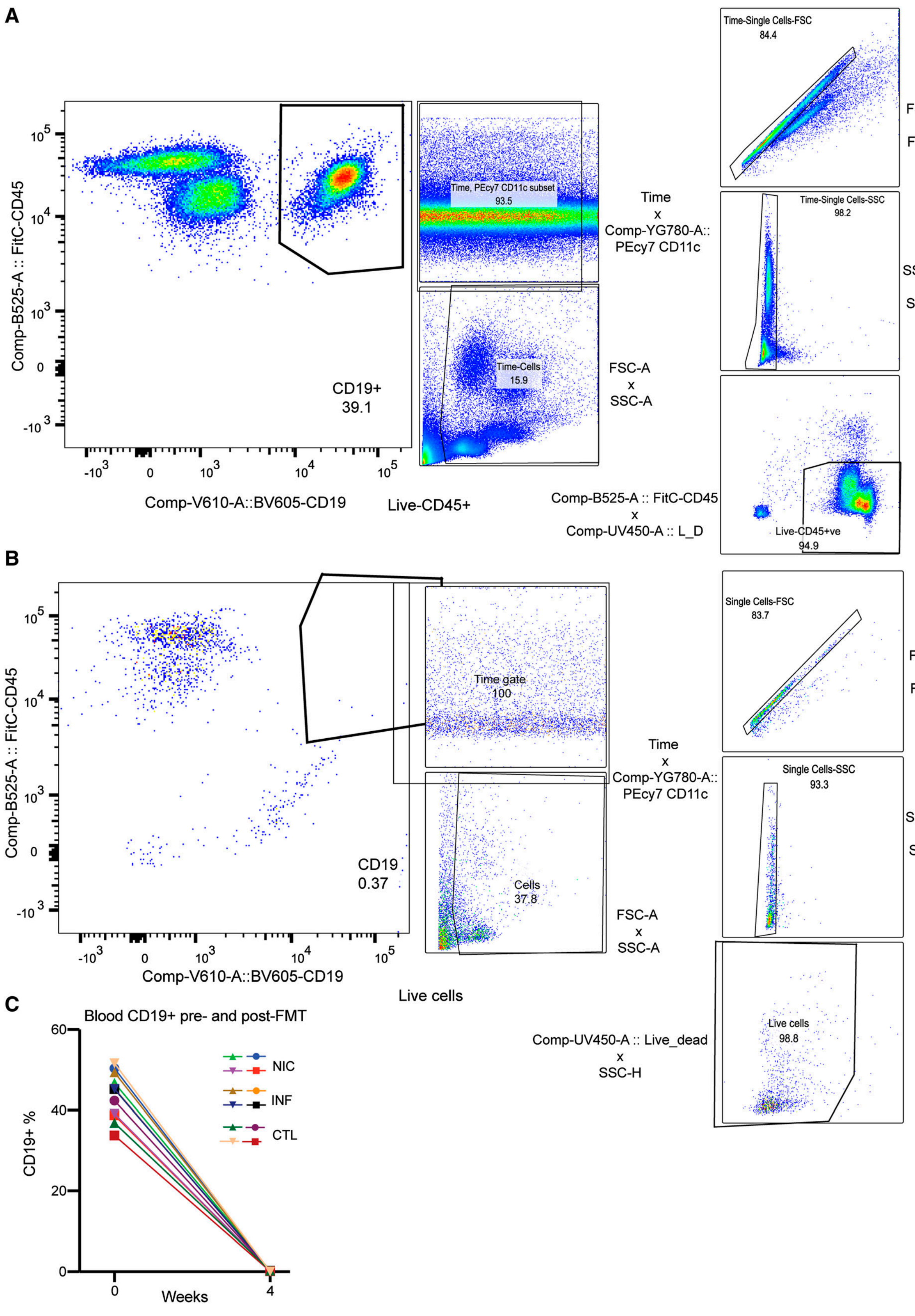

Figure S3. **Flow Cytometry for mice whole blood before and after B-cell depletion. (A and B)** Gating strategy and flow cytometry data on whole blood from the same mouse at baseline **(A)** and 6 wk following anti-CD20 treatment (B). **(C)** B cells in whole blood from mice treated with anti-CD20. Representative of one experiment, n = 4 mice/group. All male mice, ages 8–12 wk.

Downloaded from http://rupress.org/jhi/article-pdf/1/1/e20250040/1943074/jhi_20250040.pdf by Joud Hajjar on 01 September 2025

https://doi.org/10.70962/jhi.20250040

JHI

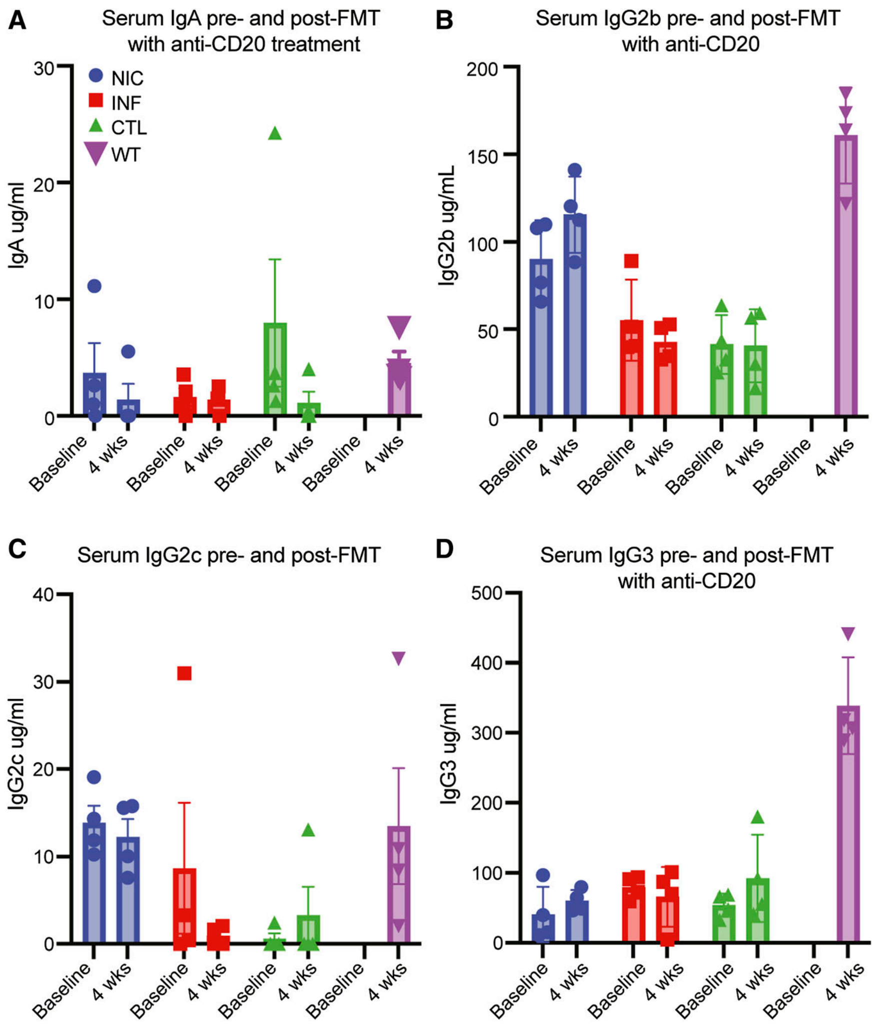

Figure S4. **Ig levels in FMT-recipient mice following anti-CD20 treatment. (A–D)** (A) IgA levels, (B) IgG2b levels, (C) IgG2c, and (D) IgG3 remained low after treatment with anti-CD20. Representation of one experiment, *n* = 4 mice/group. All female mice, ages 8–12 wk. Sera from wild-type (WT) (C57BL/6J) female mice housed in a specific-pathogen-free facility at BCM were used for comparison.

Provided online is Table S1. Table S1 shows demographics of household CTLs.

Downloaded from http://rupress.org/jhi/article-pdf/1/1/e20250040/1943074/jhi_20250040.pdf by Joud Hajjar on 01 September 2025
